# Supplementary material for: Are carbon emissions trading and green financial instruments synergistic? -Comprehensive quantitative research based on content analysis
Source: PLoS One. 2024 Mar 7;19(3):e0298601. doi: 10.1371/journal.pone.0298601 (PMC10919601; doi:10.1371/journal.pone.0298601)
Supplement: S1 Data — (ZIP) [file pone.0298601.s001.zip › Data/地级市Z_F工作报告.zip!/╜¡╬≈/╕╙╓▌╩╨/└┤╫╘╣1⁄2╓┌║┼í╛┬φ┐╦╩2╛▌═°í┐_2014.docx]

各位代表：

现在，我代表市人民政府，向大会作政府工作报告，请予审议，并请市政协委员和列席会议的同志提出意见。

一、2013年工作回顾

一年来，在省委、省政府和市委坚强领导下，全市上下按照市委四届四次全会部署，以贯彻落实《国务院关于支持赣南等原中央苏区振兴发展的若干意见》（以下简称《若干意见》）为契机，锐意进取，扎实工作，赣南苏区振兴发展开局良好。主要经济指标增速快于全国、全省平均水平。全市实现地区生产总值1673.31亿元，增长10.5 %，人均生产总值首次突破3000美元；财政总收入280.2亿元，增长21.4%；地方公共财政预算收入184.37亿元，增长30.5%；固定资产投资1330.87亿元，增长28.5%；社会消费品零售总额559.99亿元，增长13.7%；城镇居民人均可支配收入20566元，增长10%；农民人均纯收入6014元，增长13.5%。财政总收入过10亿元的县（市、区）达到9个。

（一）《若干意见》政策效应逐步释放，振兴发展开局良好。争取国务院及国务院办公厅出台8个配套文件，38个部委出台43个具体实施意见或支持政策。139项西部大开发政策或标准执行到位。39个中央国家机关及有关单位对口支援赣州18个县（市、区）。29个省厅（局）出台实施意见或签订战略合作协议。重大项目强力推进。193个市属重点工程完成投资747.7亿元。赣龙铁路扩能改造、赣韶铁路建设，寻乌至全南、南昌至宁都高速公路建设等项目顺利推进。黄金机场机坪扩建工程开工建设，昌吉赣客专获国家发改委批准立项。寻乌长宁220千伏等6个输变电工程投入运营，西气东输三线工程赣州段开工建设。平台建设稳步实施。赣州综合保税区正式批复，新增龙南、瑞金两个国家级经济开发区，国家脐橙工程技术研究中心、国家离子型稀土资源高效开发利用工程技术研究中心获批，龙南发光材料及稀土应用高新技术产业化基地被认定为国家高新技术产业化基地。

（二）产业转型升级成效明显，振兴发展支撑力持续增强。规模以上工业完成增加值635.78亿元，增长13.2%；实现主营业务收入2575.33亿元，增长21.7%。新增规模以上工业企业164家，总数达1018家。中烟赣州卷烟厂60万大箱卷烟等60个投资超亿元项目竣工投产。赣州稀土集团被确定为组建国家级大型稀土企业集团的牵头企业。稀土矿山和冶炼分离企业整治整合成效显著。稀土、钨及其应用产业主营业务收入近800亿元。战略性新兴产业完成增加值201.65亿元，增长14.9%。全市工业园区实现主营业务收入2165.54亿元，增长17.6%。金融机构人民币贷款余额1592.92亿元，增长24.14%；存贷比达60.7%，比上年末提高4.14个百分点。企业上市取得重大突破，毅德控股、康尔国际分别在香港、马来西亚上市。综合商贸物流园一期基本完工。全市接待旅游总人数2591.18万人次，增长20.7%。粮食总产量283万吨，实现“十连丰”。生猪、蔬菜等主要农产品产量稳定增长，新建服务中心城区商品蔬菜基地5248亩。油茶、花卉苗木、烟叶等种植面积不断扩大。全力组织防控柑橘黄龙病，创新举办第五届中国赣州国际脐橙节（赣南脐橙网络博览会），脐橙总产量达150万吨，实现增产增收。新增年销售收入500万元以上农业龙头企业38家、超亿元企业4家。

（三）改革开放深入推进，振兴发展活力进一步激发。南康撤市设区、部省共建教育改革发展试验区获批。我市被列为国家旅游扶贫试验区、稀土矿产地储备试点城市、全国第三批餐厨废弃物资源化利用和无害化处理试点城市。章贡区入选国家首批信息消费试点城市。稀土开发利用综合试点、低丘缓坡荒滩等未利用地开发利用试点和工矿废弃地复垦利用试点、“西果东送”农产品现代流通综合试点、国家低碳城市试点等扎实推进。重大招商活动成果丰硕。第六届海峡两岸客家高峰论坛暨第十一届赣台经贸文化合作交流大会签约项目22个，总投资15.58亿美元；中国光彩事业赣州行活动签约项目113个，总投资1040.9亿元；央企入赣投资合作洽谈会赣州签约项目41个，总投资1091.4亿元。全市实际利用外资11.07亿美元，增长13.3%；实际利用省外5000万元以上项目资金454.15亿元，增长20.4%。外贸出口总额29.16亿美元，增长2.6%。非公有制经济实现增加值1053.8亿元，增长12.5%，占生产总值比重达63%，上缴税金占财政总收入比重达72.3%。

（四）城乡统筹步伐加快，振兴发展承载力显著提升。全市城镇化率达42.56%，同比提高1.4个百分点。中心城区建成区面积94.97平方公里，新增6平方公里；总人口90.2万人，新增12.2万人。《赣州都市区总体规划》获省政府批准实施。中心城区开工重点城建项目93个。章江新区主干道路成环成网，打通一批断头路， “四桥九路一公园”项目加快推进，火车站站前广场改造等一批项目竣工。郁孤台历史文化街区整治修缮扎实推进，治脏、治乱、治堵工作巩固提升。各县县城建设加快。高标准启动17个示范镇建设。开展1847个和谐秀美乡村点建设。8个乡村入选全国首批“美丽乡村”创建试点。

（五）保障和改善民生力度加大，振兴发展成果惠及于民。全市民生类支出271.6亿元，占财政总支出56.5%。百件民生实事基本完成。改造农村危旧土坯房30万户，解决85.36万农村人口饮水安全问题，累计实现35.11万低电压用户正常用电。新建农村公路2500公里，完成危桥改造160座。改造农村中小学危房30.3万平方米，基本消除D类危房。完成电视“户户通”7.7万户，基本解决农村看电视难问题。新建保障性住房2.22万套，完成城市棚户区改造1.23万户。中心城区新增公交车100台、出租车200台。城镇新增就业9.14万人，新增转移农村劳动力17.68万人。编制实施《赣州建设全国革命老区扶贫攻坚示范区发展规划》，扎实推进罗霄山（赣州）片区扶贫攻坚，实施整村推进扶贫项目2803个，移民搬迁22061人。建立城乡低保补助标准自然增长机制，提高高龄老人补贴标准。

各项社会事业协调发展。科技创新“六个一”工程深入实施，争取国家级科技计划项目32项。赣州职教园区和乡镇公办幼儿园建设扎实推进。赣南教育学院改制升格为赣州师范高等专科学校，江西理工大学成为省部合作共建高校。国家公共文化服务体系示范区正式命名，国家印刷包装产业基地和国家级客家文化（赣南）生态保护实验区获批。《八子参军》获第十四届“文华优秀剧目奖”和五个单项奖。新型农村合作医疗惠及人群不断扩大，国家基本药物制度覆盖公办基层医疗卫生机构和行政村卫生室。十四届省运会筹备工作进展顺利。人口自然增长率6.93‰。居民消费价格比上年上涨2.4%。全面完成污染物减排任务。空气质量稳定达到国家二级标准，主要河流断面水质达标率保持在90%以上。完成造林69.16万亩。城市生态环境竞争力进入全国前20强，获评“2013中国最具生态竞争力城市”。

深入推进“送政策、送温暖、送服务”工作全覆盖常态化。安全生产和食品药品安全形势保持稳定。加强预拌商品混凝土质量监管体系建设，工程建设质量安全管理水平提升。社会治安防控体系不断完善。连续五届获“全国社会管理综合治理工作优秀市”；连续三届荣获全国综治最高奖“长安杯”。信访维稳工作得到加强。国防动员和后备力量建设水平持续提高，双拥共建活动深入开展。

一年来，政府自身建设得到加强，服务振兴发展能力稳步提高。扎实推进市直（驻市）单位绩效管理。精简审批事项335项，精简率56.11%；行政审批项目办结期限平均在9个工作日以内，缩短47%。公共资源交易市场突出问题集中整治成效明显。重大决策和政策文件合法性审查、行政复议和行政执法监督工作进一步加强。自觉接受人大、政协、民主党派、人民群众和舆论监督，办理人大代表建议、政协委员提案共505件，办复率、满意和基本满意率均达100%。认真贯彻落实中央“八项规定”和省、市有关规定，政府作风进一步改进，精文减会力度加大，以市政府和市政府办公厅名义下发的文件减少42.6%。“三公”经费支出同比有较大幅度减少。党风廉政建设和反腐败工作取得新成效。

各位代表，这些成绩的取得，是党中央、国务院和省委、省政府深切关怀，国家有关部委和省直部门倾力支援的结果，是以文清同志为班长的市委科学决策、正确领导、率先垂范的结果，是市人大和市政协监督支持，全市干部群众共同努力的结果。在此，我代表市人民政府，向全市广大工人、农民、知识分子、干部和历任老领导、老同志，向各民主党派、工商联、无党派和社会各界人士，向驻市部队、武警官兵和公安干警，致以崇高的敬意！向所有关心、支持赣州发展的同志们、朋友们表示衷心的感谢！

回顾过去一年工作，我们也清醒地看到，我市经济社会发展还存在不少困难和问题。主要是：经济总量偏小，人均水平低，自身“造血”功能不强；经济结构不合理，出口、消费增长动力不足，工业投资不够，新兴产业规模较小；要素制约仍然严重，实体经济运行困难，经济下行压力较大；重大基础设施建设滞后，城镇化水平偏低，中心城区辐射带动能力不强，农村基础设施不够完善；群众就业、住房、增收等方面还存在较大压力，改善民生、全面建成小康社会的任务仍然艰巨；政府作风和效能建设与人民群众期待还有差距。我们将采取有效措施，切实加以解决。

二、2014年工作任务

2014年是贯彻落实党的十八届三中全会精神、全面深化改革的开局之年，也是全面实施《若干意见》的攻坚之年。尽管宏观经济形势依然错综复杂，但经济发展长期向好的基本面没有变。党的十八届三中全会对全面深化改革作出总体部署，改革红利将逐步释放，发展活力将持续迸发。特别是随着《若干意见》的深入实施，中央和省支持力度持续加大，各类政策叠加效应加速显现，我市正处于黄金发展期。我们将按照既定的发展理念、发展思路和发展举措，一张蓝图画到底，一以贯之抓落实。

今年政府工作的总体要求是：全面贯彻落实党的十八大、十八届三中全会和习近平总书记系列重要讲话精神，以及省委十三届八次全会和市委四届五次全会精神，牢牢把握“稳中求进、改革创新”核心要求，按照省委“发展升级、小康提速、绿色崛起、实干兴赣”战略部署，坚持发展为先、生态为重、创新为魂、民生为本，抓改革、促发展、惠民生、优生态、保稳定，全力推进赣南苏区振兴发展提速提质提效，为建设创业、宜居、平安、生态、幸福赣州，与全国同步全面建成小康社会奠定扎实基础。

今年全市经济社会发展主要目标是：生产总值增长10.5%以上；固定资产投资增长22%；财政总收入增长16%，地方公共财政预算收入增长17%；规模以上工业增加值增长14%；社会消费品零售总额增长14%；实际利用外资增长10%；外贸进出口总额增长5%，其中出口增长5%；城镇居民人均可支配收入增长10%；农民人均纯收入增长12%；人口自然增长率控制在7.6‰以内；城镇登记失业率控制在4.5%以内；居民消费价格总水平涨幅控制在3.5%左右；完成省政府下达的节能减排计划任务。

重点做好九个方面的工作。

（一）着力推进改革创新

按照《市委贯彻落实〈中共中央关于全面深化改革若干重大问题的决定〉的意见》，抓好重点领域和关键环节的改革。

切实转变政府职能。优化政府机构设置、职能配置、工作流程，三季度基本完成市、县政府机构改革任务。加快事业单位分类改革。出台政府购买公共服务办法，制定政府购买公共服务目录。深化行政审批制度改革，认真做好行政审批事项 “接、放、管”工作，最大限度将行政审批事项下放到县（市、区），并切实加强下放事项后续监管。推进行政审批流程再造，提高行政效能。推广南康依托电子政务平台加强政务公开和政务服务工作试点经验。利用信息化手段，推进行政审批事项网上并联审批，公开目录清单，实施电子监察，实现公开公平“阳光审批”。

加快财税体制改革。深入推进预算管理制度改革，完善全口径财政预算编制体系。做好财政预决算信息以及“三公”经费公开工作，将县（市、区）信息公开试点覆盖面扩大到50%以上。强化财政资金绩效管理。按5%的比例压减“三公”经费等一般性支出预算。推进“营改增”税制改革。争取国家在赣州开展稀土资源税改革试点，争取落实稀土和钨废弃物纳入资源综合利用税优惠政策目录。建立权责发生制的政府综合财务报告制度，加强政府债务管理，防控债务风险。

深化金融改革创新。加快区域性金融中心建设，优化金融生态环境。大力发展小额贷款、融资性担保、金融及融资租赁、汽车及消费金融、投资基金等创新型机构。加快农信社改制农商行步伐，积极开展网络小额贷款公司、民间资本管理公司等创新试点，争取民营银行试点。探索建立金融资产交易等平台。加快重组稀有金属交易所。支持企业上市融资和发行债券，推动一批中小企业对接“新三板”挂牌融资。推广“小微信贷通”、“财园信贷通”，充分发挥金盛源担保有限公司作用。扩大农业保险覆盖面，积极发展脐橙等大宗农产品保险。

促进多种所有制经济共同发展。加快国企改革，做大做强城投集团，将发投集团、国有资产经营公司改组为国有资本投资公司，组建市级产业引导股权投资基金公司。按照 “非禁即入”原则，大力促进非公有制经济更好更快发展。鼓励引导民间投资进入基础设施、公用事业等领域，鼓励非公有制经济参与国企改革，大力发展国有资本、集体资本和非公有资本等交叉持股、相互嫁接、多元融合的混合所有制经济。整合政府公共资源交易机构，建立公共资源集中交易平台，逐步推进稀有金属、金融资产、环境能源产品、特许经营权等公共资源及股权、债权交易。

推进农村产权制度改革。抓好农村集体土地所有权、农村集体建设用地使用权、宅基地使用权的确权登记发证工作。在先行试点的基础上，全面开展农村土地承包经营权确权登记发证工作及农民住房登记发证工作。加强县、乡、村农村土地流转服务管理体系建设。通过土地托管、代耕和股份合作等形式，有序引导土地承包经营权向专业大户、家庭农场、农民合作社、农业企业流转。积极探索农村集体产权股份制改革，增加农民集体资产收益。创新农村金融服务机制，推动果园经营权、林权、土地承包经营权抵押担保贷款，吸引金融资本推进现代农业发展。

（二）充分释放《若干意见》政策效应

加快《若干意见》明确的重大政策落实落地，争取在全面落实差别化产业目录等西部大开发政策、申报“较大的市”、出台央企对口帮扶实施意见等方面取得新突破，构筑振兴发展新优势。

抓好对口支援工作。制定完善对口支援有关规划和工作方案，突出援县促市，“点、线、面”结合，积极争取中央国家机关及有关单位更多新政策、新措施落地。紧紧抓住对口支援的难得历史机遇，充分发挥中央国家机关及有关单位的独特优势，推动县域经济发展大跨越。落实国家主体功能区规划，支持中心城区近郊县、产业强县、资源富县发挥比较优势，积极参与区域产业分工，壮大主导产业，形成特色鲜明、主业突出、实力较强、错位发展的县域经济板块。支持有条件的县（市）开展县域经济科学发展体制创新试验，完善县域经济科学发展综合考核评价体系，促进县域经济在全省进位赶超。力争财政总收入过10亿元的县（市、区）新增3个，2个县（市、区）突破30亿元。

加快重大基础设施项目建设。争取《赣闽粤原中央苏区振兴发展规划》、《赣州市区域性综合交通枢纽规划》等尽快批复实施。做好赣深客专、鹰梅铁路、赣井铁路、华能瑞金电厂二期、神华集团发电项目和煤炭储备周转基地等重大项目申报及前期工作。开工建设昌吉赣客专，建成赣韶铁路，加快赣龙铁路扩能改造。推进黄金机场改扩建、赣州东（红都）500千伏输变电等工程，加快兴国至赣州、寻乌至全南、南昌至宁都、宁都至定南、广昌至吉安（赣州段）等高速公路建设。

推进先行先试和试点示范。用好用足用活《若干意见》赋予的先行先试权，抓好部省共建赣州教育改革发展试验区、国家旅游扶贫试验区、稀土开发利用综合试点、稀土矿产地储备试点、低丘缓坡荒山等未利用地开发利用试点和工矿废弃地复垦利用试点等试点事项推进落实，积极创建国家生态文明先行示范区，力争更多改革在赣州探索实践。争取瑞兴于经济振兴试验区规划早日获批并启动实施。抓紧国家电子商务示范城市、现代物流技术应用和共同配送综合试点城市、服务外包示范城市等申报工作。

（三）打好工业强攻战

坚持市场导向，集聚要素资源，着力提高工业投资比重，培育壮大产业集群，把赣州经济大厦建立在坚实的工业基础之上。

着力做强主导产业。围绕培育稀土、钨及其应用和新能源汽车及其配套、铜铝有色金属三个主营业务收入分别超1000亿元的优势产业集群，加快产业发展升级。以加快国家级稀土大型企业集团建设为契机，继续加大资源整治整合力度，推进稀土、钨及其应用产业向高端延伸，重点发展稀土永磁材料和机电应用产业，稀土发光材料及应用产业，稀土储氢材料及动力电池产业，硬质合金、硬面材料及数控刀具、切削刀具和工程工具产业，力争稀土、钨及其应用产业主营业务收入突破千亿元。充分发挥国家支持国内整车企业在赣州设立分厂的特殊政策优势，加快引进整车生产企业，加快新能源汽车及其配套企业聚集，加快中国汽车零部件（赣州）产业基地建设。推进铜铝有色金属循环经济产业园建设。做强南康家具品牌，做大赣南家具产业。支持以青峰药业为龙头的生物制药产业裂变发展。推动电子信息、现代轻纺、食品工业、氟盐化工、新型建材等产业上新台阶。积极培育新能源、节能环保等产业。支持县（市、区）培育主营业务收入过百亿元产业，帮助县（市、区）加速产业集聚，培植各具特色的主导产业。

着力培育企业“航母”。推动存量资产和增量投入向优势产业、优强企业、优秀企业家聚集，促进优势资源向成长好、技术新、贡献大的企业倾斜配置，打造一批主营业务收入超50亿元、100亿元的龙头企业。围绕形成与龙头企业发展成龙配套的中小微企业集群，建立中小企业公共服务平台，大力扶持“专、精、特、新”中小企业健康发展。健全完善帮扶企业发展工作机制，切实解决企业招工难、用工贵，融资难、融资贵，以及水、电、气价偏高等问题，降低营商成本。推进省级工业园区调区扩区，提高园区土地利用率和投资强度。加快建设征信融资体系示范区和融资超市，提升园区基础设施和生活设施配套水平，为企业发展打造集聚平台，营造良好环境，加速企业规模扩张。

着力实施创新驱动。坚持以“升”促建，争取赣州高新技术产业园区升格为国家高新技术产业开发区。加大科技投入，建立科技创新投资引导基金。加快国家离子型稀土资源高效开发利用工程技术研究中心、国家脐橙工程技术研究中心等国家级技术创新平台建设。支持章源钨业国家级企业技术中心、青峰药业博士后科研工作站建设，新建一批产业技术开发载体、企业技术中心和博士后工作站，支持有条件的龙头骨干企业联合高校院所建立产业技术创新战略联盟，促进重大科技成果集成和转化。以江西理工大学为依托，联合中国工程院等国内相关院所和大型企业集团，加快建设国家钨与稀土战略性新兴产业协同创新中心。高度重视信息化规划与建设，推动信息技术研发、产业发展和应用需求良性互动，促进信息化与工业化深度融合。

（四）加快以人为核心的新型城镇化

坚持以人为本、优化布局、生态文明、传承文化的基本原则，着力加快城镇化步伐，提高城镇化质量。

推进农业转移人口市民化。统筹解决已经转移到城镇的农村人口落户、住房、就业和权益保障等问题。加快户籍制度改革，探索流动人口居住证制度。让农民工随迁子女在流入地免费接受义务教育，并纳入普惠型学前教育和免费中等职业教育。加快赣州职教园区建设，打造国家级综合性职业技能培训基地，对农民工实行免费培训。把进城落户农民纳入城镇住房保障体系，在进城务工人员集中的工业园区建设公租房。

优化城镇化布局和形态。科学实施《赣州都市区总体规划》，启动控制性详细规划、各专项规划编制工作。制定出台《推进新型城镇化发展的实施意见》，以中心城市为龙头，瑞金、龙南市域副中心城市为依托，京九、厦蓉综合走廊为两轴，县城和示范镇、中心镇为节点，构建经济充满活力、生活品质优良、生态环境优美的赣南城镇体系。加快建设章康新区，有序完成南康撤市设区管理体制调整，推动赣县撤县设区、龙南撤县设市。推动瑞金、龙南依托国家级经济开发区，壮大城市体量，完善城市功能，提高城市品质。推动各县县城扩容提质，抓好示范镇、中心镇建设。树立“全域规划”理念，推动城乡一体化规划，加强城乡规划监督管理。

加强中心城市建设和管理。着力提高中心城区首位度，促进赣县、上犹、崇义与中心城区同城发展。继续推进“四桥九路一公园”等重大城建项目建设，打通章江新区剩余路网和河套老城区断头路，完善章江新区生活配套设施，加强市政设施管养。完成郁孤台历史文化街区整治工程、省运会体育场馆建设，实施南市街历史文化街区保护整治工程。依托现有山水脉络等独特风光，融入赣南客家建筑文化元素，让居民望得见山、看得见水、记得住乡愁。加快构建以公共交通为主的出行系统，加密和优化中心城区公交线路，力争实现公共交通站点500米全覆盖，开通至瑞金、龙南的城际公交。启动上犹江引水工程建设。加快建设第二垃圾处理场。新建和改造提升一批学校、菜市场、公厕、停车场、垃圾中转站，建成一批小游园、小广场。加强城市地下空间开发利用，加快城市地下管网、综合防护体系建设。完善城市治理结构，巩固省级卫生城市成果。

促进产城互动融合。积极开展现代服务业综合改革试点，力争在区域性金融、物流、旅游三大中心建设上取得更大突破。加快综合商贸物流园、空港物流中心建设，构建电商物流网络，推动多式联运，降低物流及通关成本。积极发展报刊传媒、影视制作、动漫游戏和文化创意等文化业态，推进印刷包装产业基地、红色影视基地建设。加快旅游基础和服务设施建设，推动瑞金共和国摇篮景区创建国家5A级景区，培育多元旅游新业态。整顿规范建筑市场秩序，促进房地产市场平稳健康发展。大力发展信息消费、电子商务、医疗保健等新型服务业，培育消费热点，扩大消费需求。

（五）扎实做好“三农”工作

加快构建新型农业经营体制机制，发展现代农业，促进农业基础稳固，农村和谐稳定，农民安居乐业。

培植壮大优势特色产业。严守耕地保护红线，稳定粮食生产，提高粮食单产水平，确保粮食总产280万吨以上。大力发展脐橙、生猪、蔬菜、油茶、花卉苗木、工业原料林等主导产业，培育壮大茶叶、烟叶、白莲、家禽、水产等区域特色产业，提升特色农产品精深加工水平。新增服务中心城区规模蔬菜基地5000亩。推动赣南脐橙产业扬品牌、调结构、稳面积、提品质、增效益。抓好重大动植物疫情防控，全力打好柑橘黄龙病防控攻坚战。加强农产品质量安全监管体系和能力建设，确保“舌尖上的安全”。支持每个县（市、区）建设1个以上现代农业示范园区。大力发展无公害农产品和有机食品，建立农产品质量可追溯制度。

创新农业经营体制机制。重点加快培育家庭农场、专业大户、农民合作社、农业企业等新型农业经营主体，提高农业集约经营、规模经营、社会化服务水平。新增农民合作社600家、入社农户5万户，新增家庭农场300家。鼓励发展混合所有制农业产业化龙头企业，推动集群发展。新增规模以上农业产业化龙头企业35家。抓好国家级赣南脐橙专业市场建设，加快农村农产品市场和物流体系建设。提高农民素质，培养造就新型农民队伍。

完善农业农村基础设施。加强以水利为重点的农业基础设施建设，建设一批高标准农田。加快灌区续建配套与节水改造，大力推进小农水重点县建设，因地制宜建设一批“五小”水利工程。坚持镇村联动发展，推进和谐秀美乡村建设，打造一批人口集中居住、产业集聚发展、功能集成配套的新农村综合体，一批组团式、生态化的和谐秀美社区。

（六）提升开放型经济水平

创新开放思路，拓展开放空间，优化开放环境，打造开放型经济升级版。

扩大招商引资实效。继续推进央企入赣。改进招商方式，大力推进专业队伍招商、产业化招商、市场化招商、网络招商。紧盯央企、大型民企等目标企业，引进行业领军企业、产业链“链核”企业和关键配套企业。开展重大招商项目落地年活动，推动央企入赣投资洽谈会、第十一届赣台经贸文化合作交流大会、光彩事业赣州行签约项目落地。坚持引资、引技、引智相结合，出台具体政策，加大高端装备、高端人才引进力度，鼓励赣商、在外务工人员返乡创业。

加快开放平台建设。推进赣州开发区和龙南、瑞金经济开发区扩区增容，争取设立 “三南”承接加工贸易转移示范地。构建将沿海口岸“延伸”到赣州的新型内陆口岸服务体系，推进赣州“无水港”、航空港及定南、龙南、瑞金口岸作业区建设，实现进出口集装箱全直通。发挥赣州至厦门“五定”班列效应，争取开通赣州至深圳“五定”班列。增加或加密至国内主要城市航线。

促进全方位开放合作。加强与珠三角、海西经济区、长三角地区对接合作，积极融入21世纪海上丝绸之路建设。推进赣南承接产业转移示范区和赣闽、赣粤产业合作区建设。加快赣州综合保税区建设，积极改革创新，探索负面清单管理模式，着力打造港区一体的自由贸易创新试验区、内陆“大通关”示范区和中部地区金融改革开放“桥头堡”。鼓励支持优势企业“走出去”开拓国际市场。

（七）加强生态文明建设

守住生态红线，发掘生态效益，发展绿色经济，构筑我国南方地区重要的生态屏障，让赣州成为天更蓝、地更绿、水更净的美好家园。

加强生态环境保护。大力实施净空、净水、净土工程，创建国家级森林城市。加强污染物特别是大气污染防治，在中心城区开展PM2.5监测，保持城市环境空气质量达到国家二级标准。强化重点流域特别是城市饮用水源地水污染防治，全面排查整治章江、贡江、东江流域污染企业，加强章贡区水西污染源综合治理。继续抓好废弃矿山地质环境恢复治理、重金属污染治理、崩岗治理和土壤修复。推进农业面源污染治理。加强垃圾无害化处理、城镇生活污水集中处理及工业污水处理。

大力发展生态经济。开展好全国低碳城市试点，大力发展森林碳汇，切实保护生态公益林。抓好赣州环境能源交易所、中国南方（赣州）林业产权交易中心运营。加快赣州餐厨废弃物资源化利用和无害化处理试点。推进赣州开发区等园区循环化改造，建设国家生态工业示范园区。引导河流源头县和重点生态保护区调整产业结构，重点发展生态旅游、绿色农业、健康服务业，加速把生态优势转化为产业优势、经济优势和竞争优势。

构建生态文明制度。整合重组森林资源资产，组建林业集团。逐步推广合同能源管理机制，探索环保服务总承包和环境治理特许经营，推进环境污染第三方治理。探索建立生态补偿和生态环境共建共享机制，争取将东江源、赣江源列入国家生态补偿试点，贡江源纳入国家重点生态功能区。制定生态文明评价指标体系，加大资源消耗、环境损害、生态效益等考核指标权重，用制度保护好青山绿水、美丽生态。

（八）努力增进民生福祉

推进全国革命老区扶贫攻坚示范区建设，继续着力解决突出民生问题，积极回应人民群众关切，让发展成果更多更公平惠及全市人民。

扎实推进民生工程。筹集资金200亿元以上，在完成省下达50件民生工程实事基础上，办好40件民生实事。完成剩余14.28万户农村危旧土坯房改造，解决剩余40.5万农村人口饮水安全问题；基本完成农村中小学校舍危房改造；加快农村电网改造；新建农村公路2800公里，改造危桥100座。完善住房保障体系，开工建设公租房1.56万套，改造各类棚户区1.46万户，加快中心城区返迁房建设。积极促进就业创业，完善社会保险补贴、小额担保贷款等就业政策体系。统筹城乡居民医保，建立城乡居民大病保险。完善城乡低保制度，提高城乡低保、农村五保户供养和优抚对象等生活标准，新增农村低保对象4.2万人。推进社会养老服务体系建设。着手规划残疾人集中安居、就业和康复的“康乐工程”。着力实施《赣州建设全国革命老区扶贫攻坚示范区发展规划》。完成2.5万贫困人口移民搬迁，抓好整村推进扶贫工作。推进精准扶贫，设立扶贫救助专项基金。深入实施“雨露计划”和“一户一名技能工”培训工程，免费为贫困家庭培训技能工。

加快发展社会事业。促进公办学校标准化建设，推进义务教育均衡发展。启动教育云示范工程，促进优质教育资源共享。推动乡镇公办幼儿园建设。健全家庭经济困难学生资助体系。加快农村中小学教师保障性住房建设。建设国家公共文化服务体系示范区和国家级客家文化（赣南）生态保护实验区，打造红色文化传承创新区。推进广播电视“户户通”工程。加强赣南苏区革命遗址保护，完成零散纪念设施抢救保护工程。坚持计划生育基本国策，依法落实“单独两孩”生育政策。巩固和完善国家基本药物制度。稳妥推进公立医院改革，深化基层医疗卫生机构综合改革，完善农村三级医疗卫生服务网络。加快章江新区新人民医院和市儿童医院建设。实行最严格的食品药品监管制度。向社会全面开放公共体育场馆，承办好第十四届省运会。争创国家文明城市。深化双拥创建工作，加强国防后备力量建设。基本完成第三次全国经济普查工作任务。

维护社会和谐稳定。坚持系统治理、依法治理、综合治理和源头治理，着力推进政府治理体系和治理能力现代化。深入推进“送政策、送温暖、送服务”工作。建立完善社会矛盾预防化解、公共安全保障、网络安全管理和公平正义实现机制，完善重大决策社会稳定风险评估机制。健全基层信访工作网络，从源头上预防和化解社会矛盾。广泛开展全民普法教育，深入推进法治赣州建设。加强城乡社区建设，推行城乡社区网格化管理。完善基层社会服务体系，激发社会组织活力。落实安全生产“一岗双责、党政同责”的要求，严防重大安全事故发生。强化气象灾害监测预警、防震减灾能力建设。健全和完善突发事件应急管理体系，提高应急管理水平和应急处置能力。

（九）建设人民满意政府

始终把人民群众对美好生活的向往作为政府工作的不懈追求，以改革创新精神，进一步加强政府自身建设，切实担负起服务人民群众和振兴发展的重任。

坚持依法行政。牢固树立法治理念和法治思维，严格按照法定权限和程序行使权力、履行职责，完善行政决策机制，把公众参与、专家论证、风险评估、合法性审查和集体讨论决定，作为规范性文件和重大决策出台的必经程序，提高科学民主决策水平。主动接受人大法律监督、工作监督和政协民主监督，自觉接受舆论监督和社会监督。推进政务公开和政府信息公开，推行权力清单制度，依法公开政府权力运行流程。

坚持服务为民。扎实开展以为民务实清廉为主要内容的党的群众路线教育实践活动，集中解决“四风”问题，坚持求真务实，改进工作作风，努力为基层、企业和群众提供更加优质高效便捷的服务。规范行政服务中心建设，落实行政审批“两集中两到位”。创新政府服务管理方式，推行无纸化办公。推动政府职能向创优发展环境、提供优质公共服务、维护社会公平正义转变。

坚持清正廉洁。严格落实党风廉政建设责任制，全面推行廉能风险防控机制标准化建设，加大惩治和预防腐败力度。加强对政府投资项目资金使用、工程招标投标、土地出让开发、政府采购和国有资产转让等方面的监督管理。认真落实中央“八项规定”和省、市有关规定，规范办公用房使用管理，实施公务用车改革，厉行勤俭节约，反对铺张浪费。深入开展损害群众利益突出问题专项治理，坚决纠正部门和行业的不正之风，切实做到干部清正、政府清廉、政治清明。

各位代表，振兴发展的宏伟蓝图引领着我们，全面建成小康社会的目标鼓舞着我们，人民群众过上美好幸福生活的新期待激励着我们。让我们更加紧密地团结在以习近平同志为总书记的党中央周围，在市委的坚强领导下，展解放思想之翼，扬改革创新之帆，践真抓实干之行，务惠民利民之效，奋力开创全面深化改革、加快振兴发展新局面！
